# Development of Microfluidic Chip-Based Loop-Mediated Isothermal Amplification (LAMP) Method for Detection of Carbapenemase Producing Bacteria

**DOI:** 10.1128/spectrum.00322-22

**Published:** 2022-08-18

**Authors:** Bin Wu, Xinxin Tong, Bin Chen, Wenchang Yuan, Mingpeng Fu, Xiao Yang, Huiling Chen, Guohao Zhang, Guojun Wu, Banglao Xu

**Affiliations:** a Department of Laboratory Medicine, Guangzhou First People's Hospital, School of Medicine, South China University of Technology, Guangzhou, Guangdong, China; b Department of Blood Transfusion, Guangzhou First People's Hospital, School of Medicine, South China University of Technology, Guangzhou, Guangdong, China; c KingMed School of Laboratory Medicine, Guangzhou Medical University, Guangzhou, Guangdong, China; d Baicare Biotech Company, Beijing, China; University of Cincinnati

**Keywords:** microfluidic chips, carbapenemase-producing organisms, loop-mediated isothermal amplification, blood cultures, clinical bacteria isolates, detection performance

## Abstract

The rapid and accurate diagnostic methods to identify carbapenemase-producing organisms (CPO) is of great importance for controlling the CPO infection. Herein, we have developed a microfluidic chip-based technique to detect CPO and assessed its clinical value in detecting CPO directly from blood cultures (BCs). The detection performance of the microfluidic chip-based LAMP amplification method was analyzed retrospectively on a collection of 192 isolates including molecularly characterized 108 CPO and 84 non-CPO and prospectively on a collection of 133 positive BCs with or without CPO suspicion, respectively. In the retrospective study, the microfluidic chip-based LAMP amplification method exhibited 87.5% accuracy (95% CI [82.0–91.5]), 97.7% sensitivity (95% CI [91.2–99.6]), 78.8% specificity (95% CI [69.5–86.0]), 79.6% positive predictive value (PPV) (95% CI [70.6–86.5]) and 97.6% negative predictive value (NPV) (95% CI [90.9–99.6]). Among the 192 isolates, 22 (11.5%) false-positives (FP) and 2 (1.0%) false negatives (FN) were observed. In the prospective study, the 133 routine isolates of positive BCs including 18 meropenem-resistant CPO and 115 non-CPO were assessed, and 4 FP were observed in non-CPO and CPO, respectively. The current method showed a total detection performance of 94.0% accuracy (95% CI [88.4–97.1]), 100.0% sensitivity (95% CI [73.2–100.0]), 93.2% specificity (95% CI [86.7–96.8]), 63.6% PPV (95% CI [40.8–82.0]) and 100.0% NPV (95% CI [95.8–100.0]). In summary, the microfluidic chip-based LAMP amplification method is reliable for the rapid screening and detection of CPO with high accuracy, sensitivity, and specificity, and could easily be implemented in clinical microbiology laboratories.

**IMPORTANCE** Rapid and accurate identification of CPO may reduce the genetic exchanges among bacteria and prevent further dissemination of carbapenemases to non-CPO. The current method had designed microfluidic chip-based LAMP amplification method for multiplex detection of carbapenemase genes and evaluated the detection performance of the newly method. The current method can rapidly screen and detect CPO with high accuracy, sensitivity, and specificity, and could easily be implemented in clinical microbiology laboratories, as this will reduce the carbapenem resistance issues worldwide.

## INTRODUCTION

The rapid emergence and expansion of carbapenem-producing organisms (CPO) is an urgent global public health threat, mostly as a consequence of carbapenemase gene acquisition (1). Carbapenemase genes offer a stable and transferable form of resistance, enabling spread via clonal expansion or by horizontal transfer of genes to naive bacteria (2). Thus, simple and reliable confirmatory tests are mandatory to rapidly discriminate CPO from non-CPO. Both phenotypic and molecular-based assays are available for detection of carbapenemase production in Gram-negative bacilli including *Enterobacteriaceae*, Pseudomonas aeruginosa and Acinetobacter baumannii. Various phenotypic confirmation tests for detecting carbapenemases have been developed, including growth-based assays which measured the growth or inhibition of microorganisms in the presence of an antibiotic (for example, modified Hodge test [MHT], modified carbapenem inactivation method [mCIM]), hydrolysis methods which detect the production of hydrolysis that is catalyzed by carbapenemase enzymes (for example, Carba NP and Matrix-assisted laser desorption ionization–time of flight mass spectroscopy [MALDI-TOF MS]), and lateral flow immunoassays which detect carbapenemase enzymes through the use of specific antibodies (for example, NG-test Carba5) (3). In addition, several molecular methods such as simplex and multiplex PCRs, microarrays, DNA hybridization and sequencing are considered as the reference methods for identification of carbapenemase genes (4). Accordingly, a number of phenotypic and molecular tests have been developed for the detection of CPO directly from blood cultures (BCs), however, each approach has some weaknesses: the incubation times of different bacterial or carbapenem molecules of MALDI-TOF MS assays vary from 20 min to 4 h, which means it is difficult to standardize these protocols (5); PCR has fewer testing targets and does not meet the need for the identification of multiple drug resistance genes (6); Nucleic Acid (NA) microarray has high-throughput properties, but it is expensive and complex, which is not suitable for routine clinical samples (4); The Carba NP test need additional incubation time and often lack sensitivity and specificity (5). Therefore, developing a rapid, low-cost, high-throughput and user-friendly molecular diagnostic technique is critical for timely diagnosis and antibiotics choice of clinicians.

Bloodstream infection (BSI) is a major cause of mortality in hospitalized patients worldwide, with an overall mortality rate of close to 40% (7). Delays in initiating an effective antimicrobial therapy are associated with a 7.6% decrease in survival for patients developing septic shock (8). Conventional techniques for bacteremia diagnosis usually take at least 48 h, for this reason, empirically administered with broad-spectrum antibiotics is the most common therapy strategy of patients with BSI, which can be neurotoxic and nephrotoxic (9).

Microfluidic chips are a promising platform for microbiological assays. These so-called “micro total analysis systems (μTAS)” or “labs-on-a-chip” have gained in popularity due to their flexibility for automation, integration, miniaturization, and multiplexing (10). In the past decade, microfluidic chip handling systems with microchambers and microchannels have been developed for a range of practical applications, particularly for NA analysis in microbiological assays (11). Deployment of microfluidic chip-based detection techniques have distinct advantages, including high-throughput, portability, relatively small amount of reagent and sample usage, fast and the ability to integrate multiple elements on a single chip (12). In 2000, Notomi et al. (13) reported a novel NA amplification method called loop-mediated isothermal amplification (LAMP), that amplifies DNA with high specificity, efficiency and rapidity under isothermal conditions. Compared to the conventional molecular detection methods, LAMP was demonstrated to be quicker and more stable, sensitive, and specific for NA identification (14). In recent years, integrated microfluidic LAMP systems have been reported for multiple applications.

The aim of this study was to evaluate the detection performance of the newly designed microfluidic chip-based LAMP amplification method for multiplex detection of carbapenemase genes and its added value in routine diagnostic workflows directly from BCs. The performance of this method was retrospectively and prospectively analyzed on a collection of 192 isolates including molecularly-characterized 108 CPO and 84 non-CPO and on 133 positive BCs.

## RESULTS

### Retrospective performance analysis.

An overview of carbapenemase genes of all isolates evaluated is found in Table 1. We analyzed on a collection of 192 isolates including molecularly-characterized 108 CPO and 84 non-CPO to evaluate the detection performance of the microfluidic chip-based LAMP amplification method. This method exhibited a total detection performance of 87.5% accuracy (95% CI [82.0-91.5]), 97.7% sensitivity (95% CI [91.2-99.6]), 78.8% specificity (95% CI [69.5-86.0]), 79.6% PPV (95% CI [70.6-86.5]) and 97.6% NPV (95% CI [90.9-99.6]) (Table 2). At the same time, the detection performance of *Enterobacteriaceae* was comparable to non-fermentative bacteria, which showed 86.4% accuracy (95% CI [79.2-91.4]), 98.3% sensitivity (95% CI [89.5-99.9]), 76.1% specificity (95% CI [63.9-85.3]), 78.1% PPV (95% CI [66.6-86.6]) and 98.1% NPV (95% CI [88.4-99.9]) of *Enterobacteriaceae*, and 89.6% accuracy (95% CI [79.7-95.1]), 96.7% sensitivity (95% CI [80.9-99.8]), 83.8% specificity (95% CI [67.4-93.2]), 82.8% PPV (95% CI [65.7-92.8]) and 96.9% NPV (95% CI [82.0-99.8]) of non-fermentative bacteria (Table 2). Among the 192 isolates, 22 (11.5%) false positives (FP) and 2 (1.0%) false negatives (FN) were observed, however, 86 CPO (44.8%) and 82 non-CPO (42.7%) were correctly detected (Table S3 and S4). In addition, the current assay exhibited 80.0% accuracy (95% CI [69.5-87.2]) of single carbapenemase CPO, 79.3% accuracy (95% CI [61.3-90.5]) of multiples carbapenemase CPO and 98.8% accuracy (95% CI [92.8-100.0]) of carbapenemase negative bacteria. This showed that the detection performance of carbapenemase negative isolates showed higher detection accuracy than single carbapenemase and multiples carbapenemase isolates, and single carbapenemase and multiples carbapenemase isolates showed similar detection performance (Table S5).

**TABLE 1:** Overview of bacterial isolates evaluated by sequencing in retrospective study

	Organism	Total (n)	Carbapenemase gene							
			*bla*_KPC_	*bla*_NDM_	*bla*_VIM_	*bla*_IMP_	*bla*_oprD2_	*bla*_OXA-23_	*bla*_OXA-48_	*bla*_OXA-58_
Single carbapenemase	K. pneumoniae	21	21							
	K. pneumoniae	10		10						
	K. pneumoniae	2				2				
	K. pneumoniae	1			1					
	K. pneumoniae	1							1	
	E. coli	7	7							
	E. coli	14		14						
	A. baumannii	20						20		
	A. baumannii	1								1
	E. cloacae^a^	2		2						
Multiple carbapenemase	K. pneumoniae	5	5			5				
	K. pneumoniae	4	4	4						
	K. pneumoniae	1	1	1		1				
	K. pneumoniae	1		1		1				
	E. coli	1	1	1						
	C. koseri	2	2	2						
	A. baumannii	6	6					6		
	A. baumannii	5		5				5		
	A. baumannii	2				2		2		
	A. baumannii	1	1	1				1		
	A. baumannii	1	1					1	1	
Carbapenemase negative	E. coli	28	NA^*a*^	NA	NA	NA	NA	NA	NA	NA
	K. pneumoniae	21	NA	NA	NA	NA	NA	NA	NA	NA
	C. koseri	4	NA	NA	NA	NA	NA	NA	NA	NA
	A. baumannii	31	NA	NA	NA	NA	NA	NA	NA	NA
Total no.		192	49	41	1	11	0	35	2	1

a NA, not applicable.

**TABLE 2:** The detection performance of microfluidic chip-based LAMP amplification method on a collection of 236 clinical isolates in retrospective study

Bacteria	Total (n)	Carb pos^*a*^ (n)	Carb neg^*b*^ (n)	Accuracy [95% CI]^*c*^	Sensitivity [95% CI]	Specificity [95% CI]	PPV^*d*^ [95% CI]	NPV^*e*^ [95% CI]
Total	192	108	84	87.5 [82.0–91.5]	97.7 [91.2–99.6]	78.8 [69.5–86.0]	79.6 [70.6–86.5]	97.6 [90.9–99.6]
*Enterobacteriaceae*	125	72	53	86.4 [79.2–91.4]	98.3 [89.5–99.9]	76.1 [63.9–85.3]	78.1 [66.6–86.6]	98.1 [88.4–99.9]
Non-fermentative	67	36	31	89.6 [79.7–95.1]	96.7 [80.9–99.8]	83.8 [67.4–93.2]	82.8 [65.7–92.8]	96.9 [82.0–99.8]

a Carb Pos: Carbapenemase positive.

b Carb Neg: Carbapenemase negative.

c 95% CI: 95% confidence interval.

d PPV: Positive predictive value.

e NPV: Negative predictive value.

### Prospective performance analysis.

The performance of the microfluidic chip-based LAMP amplification method to detect and classify carbapenemases was analyzed on a collection of 133 strains directly isolated from positive BCs including 18 meropenem-resistant CPO and 115 non-CPO (Table 3). Four FP were observed in non-CPO, and 4 FP were observed in CPO including Escirichia coli carrying a KPC encoding gene, Klebsiella pneumoniae carrying a KPC encoding gene, A. baumannii carrying OXA-23, and KPC encoding genes, and A. baumannii carrying OXA-23 and NDM encoding genes (Table S6). The microfluidic chip-based LAMP amplification method exhibited a total detection performance of 94.0% accuracy (95% CI [88.4-97.1]), 100.0% sensitivity (95% CI [73.2-100.0]), 93.2% specificity (95% CI [86.7-96.8]), 63.6% PPV (95% CI [40.8-82.0]) and 100.0% NPV (95% CI [95.8-100.0]). Noteworthy, the detection performance of *Enterobacteriaceae* was better than non-fermentative bacteria, which presented 95.5% accuracy (95% CI [89.7-98.3]), 100.0% sensitivity (95% CI [70.0-100.0]), 95.0% specificity (95% CI [88.2-98.1]), 70.6% PPV (95% CI [44.1-88.6]) and 100.0% NPV (95% CI [95.2-100.0]) (Table 4).

**TABLE 3:** Overview of bacterial isolates directly from BCs detected by microfluidic chip-based LAMP amplification method

Organism	MIC(μg/mL)		Total (n)	Carbapenemase gene (microfluidic chip-based LAMP amplification method)							
	MEM	IPM		*bla*_KPC_	*bla*_NDM_	*bla*_VIM_	*bla*_IMP_	*bla*_oprD2_	*bla*_OXA-23_	*bla*_OXA-48_	*bla*_OXA-58_
K. pneumoniae	≦1	≦1	38	1	0	0	0	0	0	0	0
E. coli	≦1	≦1	45	1	0	0	0	1	0	0	0
E. aerogenes ^*a*^	≦1	≦1	2	0	0	0	0	0	0	0	0
P. mirabilis ^*b*^	≦1	≦1	2	0	0	0	0	0	0	0	0
E. cloacae	≦1	≦1	4	0	0	0	0	0	0	0	0
E. aerogenes	≦1	≦1	6	0	0	0	0	0	0	0	0
A. baumannii	≦2	≦2	17	0	0	0	0	0	1	0	0
M. morganii ^*c*^	≦1	≦1	1	0	0	0	0	0	0	0	0
E. coli	4–16	4–16	7	7	1	0	0	1	0	0	0
K. pneumoniae	≧16	4–16	6	6	1	0	0	0	0	0	0
P. mirabilis	≧16	≧16	1	1	0	0	0	0	0	0	0
A. baumannii	≧8	≧8	4	1	3	0	0	0	4	2	0
All			133	17	5	0	0	2	5	2	0

a Enterobacter aerogenes.

b Proteus mirabilis.

c Morganella morganii.

**TABLE 4:** The detection performance of microfluidic chip-based LAMP amplification method for discriminating CPO from non-CPO directly from positive BCs

Bacteria	Total (n)	Carb pos^*a*^ (n)	Carb neg^*b*^ (n)	Accuracy [95% CI^*c*^]	Sensitivity [95% CI]	Specificity [95% CI]	PPV^*d*^ [95% CI]	NPV^*e*^ [95% CI]
Total	133	18	115	94.0 [88.4–97.1]	100.0 [73.2–100.0]	93.2 [86.7–96.8]	63.6 [40.8–82.0]	100.0 [95.8–100.0]
*Enterobacteriaceae*	112	14	98	95.5 [89.7–98.3]	100.0 [70.0–100.0]	95.0 [88.2–98.1]	70.6 [44.1–88.6]	100.0 [95.2–100.0]
Non-fermentative	21	4	17	85.7 [64.5–95.9]	100.0 [19.8–100.0]	84.2 [59.5–95.8]	40.0 [7.0–82.9]	100.0 [75.9–100.0]

*^a^*Carb Pos: Carbapenemase positive.

b Carb Neg: Carbapenemase negative.

c 95% CI: 95% confidence interval.

d PPV: Positive predictive value.

e NPV: Negative predictive value.

## DISCUSSION

Resistance to carbapenem antibiotics is increasing globally and turned out to be an international public health threat. The detection of resistance gene by molecular-based technologies is the gold standard (15). It is necessary to develop a sensitive and reliable test for detection of the most frequent carbapenemase genes, with the goal of facilitating early diagnosis and controlling for the CPO infection (16). Herein, we have developed a microfluidic chip-based LAMP amplification method for the detection of 8 carbapenemase genes and evaluated the analytical performance characteristics of the newly developed method and compared the data to conventional methods.

According to the Ambler classification system, carbapenemases are commonly categorized as following Classes: Class A enzymes include the K. pneumoniae carbapenemase (KPC) family, which is commonly identified in *Enterobacteriaceae* and occasionally found in P. aeruginosa or A. baumannii; Class B enzymes include the metallo-β-lactamases (MBL), such as the New-Delhi-metallo-β-lactamases (NDM), the imipenem-resistant phenotype (IMP) family, and the Verona integron-encoded metallo-β-lactamases (VIM), which are commonly identified in *Enterobacteriaceae* and P. aeruginosa; Class D enzymes include OXA-23-like, OXA-40-like, OXA-58-like, and OXA-143-like enzymes, which are commonly reported in A. baumannii (3, 17). For most carbapenem-resistant P. aeruginosa strains, the loss of oprD (an outer membrane porin), resulting the decreased outer membrane permeability and/or an increased efflux, is the main nonenzymatic mechanisms (18). As noted above, we designed eight frequent gene variants encoding for carbapenemases including KPC, NDM, IMP, VIM, OXA-23, OXA-48, oprD2 and OXA-58 amplification of LAMP on microfluidic chips.

In the retrospective study, the high sensitivity of the current assay was observed in this study, indicated that the microfluidic chip -based LAMP amplification method is reliable for the screening and detection of CPO. Although LAMP is a specific amplification technique, in comparison with PCR (using one pair of primers), LAMP utilizes four primers to recognize six different regions of targeted sequences, which will make LAMP an excellent capability of amplifying the targeted nucleotide sequences specifically and discriminating non-target sequences efficiently (19). Unfortunately, LAMP results show nonspecific amplifications more or less, and various investigations have realized that carry-over contamination is one of the causes leading to the occurrence of false-positive results (20). This drawback of LAMP may contribute to the relatively high FP (11.5%) rate in current assay. In addition, the current study on clinical isolates showed a 79.6% PPV (95% CI [70.6-86.5]) and 97.6% NPV (95% CI [90.9-99.6]), indicating that the microfluidic chip -based LAMP amplification method was useful to rapidly differentiate between CPO and non-CPO.

The methodology developed in this study hold distinct advantages. First, the procedure for carbapenemase gene detection from clinical samples without prior cultivation of bacteria on solid medium. The most important clinical sample, which requires rapid processing in order to facilitate early diagnose and correct treatment, is blood of septic patients (21). Hence, the process of bacterial purification from BCs is crucial. The current development can provide a rapid test that reducing the turnaround time (TAT) from 24 – 48 h to 2 h for resistance detection toward certain antibiotics of relevance in a given therapeutic setting. And it also determines whether a suspected carbapenem-resistant phenotype is involved or not. Additionally, the newly developed system is cost-effective and the device is small and portable. PCR-based detection systems are widely used in fully equipped first-level hospitals, however, the high cost of the equipment and reagent hampers its application in resource-limited institutions (10). The current protocol consumes minimal amounts of samples and amplification reagents, and LAMP is a low-cost detection method only requires a constant temperature detection equipment. Therefore, it has great potential of this method for extensive clinical use. Consequently, the current assay compatible with panels comprising multiple carbapenemases genes, thus can be performed in high-throughput characteristics. It is widely known that some bacteria probably carry multiple resistance genes and may evolve into multiple antibiotic resistant populations. For therapeutic decisions of antibiotic treatment, multiple detection of carbapenem-resistant genes is important. For example, ceftazidime-avibactam (CZA), a relatively new combination of a third-generation cephalosporin and a novel β-lactamase inhibitor, has an effect against KPC-producing K. pneumoniae and MDR P. aeruginosa (22). Hence, accurate and comprehensive information of CPO detection and classification would allow the targeted antibiotic therapy.

However, there were still some limitations in our studies. Carbapenem-resistant *Enterobacteriaceae* (CRE) producing OXA-48 are mainly concentrated in European countries (France, Germany, Netherlands, Italy, the United Kingdom, and so on), Middle East (Turkey), and Mediterranean countries, including North Africa (mainly Morocco, Tunisia, Egypt, and Libya) (23). Endemicity of VIM- and IMP-type enzymes has been reported in Greece, Taiwan, and Japan, although outbreaks and single reports of VIM and IMP producers have been reported in other countries such as China, United Kingdom, Belgium and so on (24). Due to the low prevalence of bla_VIM_, bla_OXA-48_ and bla_OXA-58_ of clinical isolates in our hospital, we can't accurately evaluate the detection performance of these carbapenemase genes on the microfluidic chip-based LAMP amplification met Spectrum00322-22 hod. In addition, the current protocol in prospective study was not performed on consecutive clinical isolates but on selected some isolates with CPO suspicion representing typical isolates mostly encountered in our laboratory. This led to a relatively small number of CPO of positive BCs were included in the study.

Overall, rapid and accurate identification of CPO may reduce the genetic exchanges among bacteria and prevent further dissemination of carbapenemases to non-CPO. The current method will reduce the carbapenem resistance issues worldwide, and has good clinical application and promotion value.

## MATERIALS AND METHODS

### Bacterial isolates.

192 bacterial strains (108 *Enterobacteriaceae*, 84 non-fermentative) were obtained from archived clinical isolates at the Microbiology Unit of Guangzhou First People’s Hospital. These isolates were originally recovered between 2014 and 2020 from different specimen types. From frozen stock, each isolate was inoculated onto blood agar plates and suspended in normal saline.

### Clinical samples.

The BCs (Bactec plus/F; BD, Franklin Lakes, NJ, USA) resulting positive were collected from November 2018 to December 2020. A total of 133 positive BCs showing single bacterium growth were analyzed using the conventional laboratory diagnostic method and our newly developed microfluidic chip method. Aliquots of blood culture medium were Gram stained to confirm Gram-negative bacilli growth and to initiate subcultures to eliminate potential false positive results. This study was approved by the ethics committee of Guangzhou First People’s Hospital.

### Laboratory workflow with conventional method and microfluidic chip method upon suspicion of CPO.

**FIG 1:**
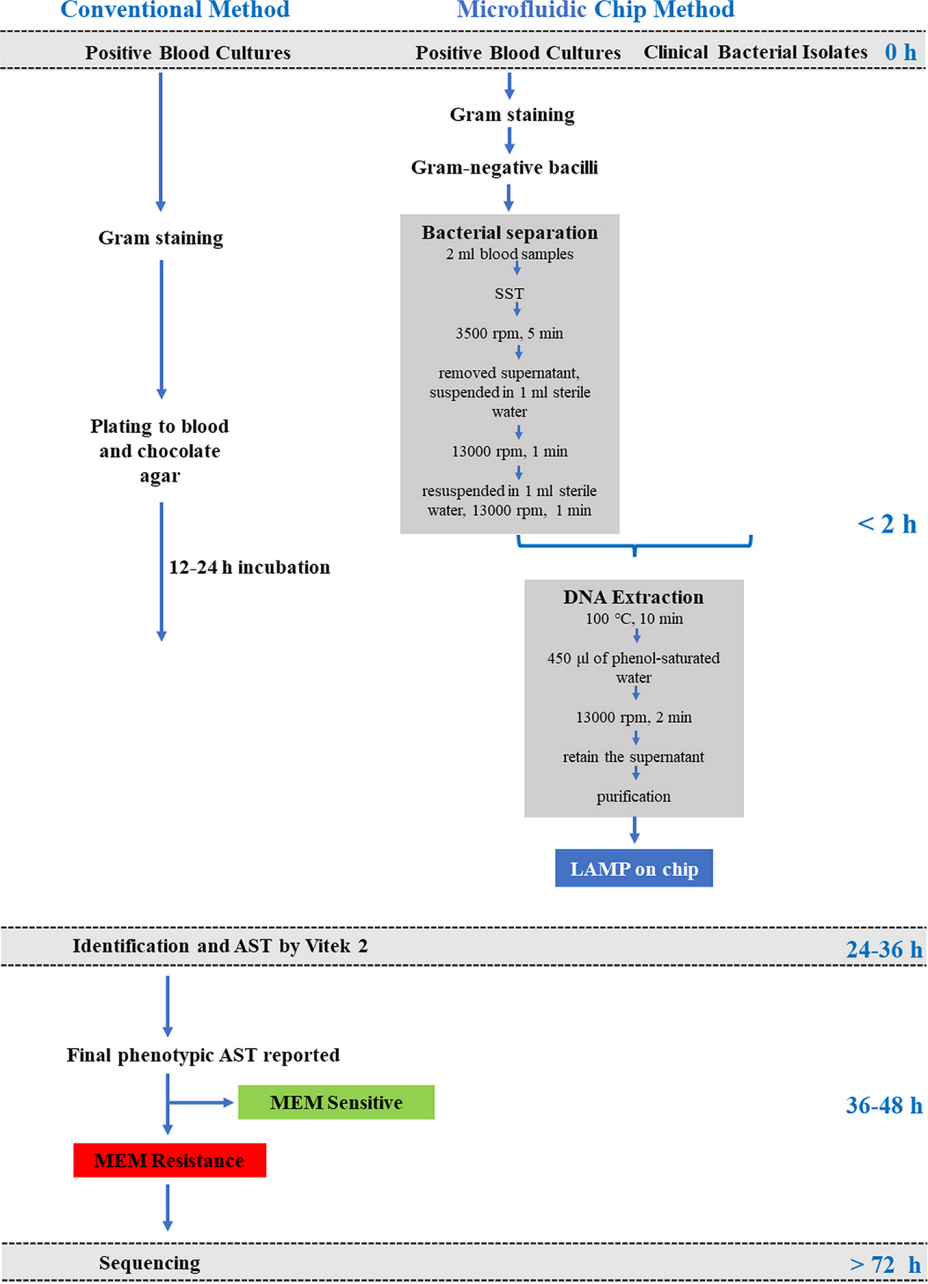
Conventional and microfluidic chip-based methods laboratory workflows.

The sequential assay workflow (Fig. 1) upon CPO suspicion consisted of (i) conventional microbiological methods and sequencing; (ii) bacterial isolation from positive BCs using serum separator gel tubes; (iii) DNA extraction of purified bacteria; (iv) carbapenemase genes detection (LAMP) on microfluidic chips.

### Conventional microbiological methods and sequencing.

When BD Bactec blood culture system showed a positive signal, gram staining was performed, followed by subculture on blood and chocolate agar plates at 35°C for 18 to 24 h. After incubation, the colonies grown on the agar plates were used for identification and antibiotic susceptibility test (AST) using the commercial automated Vitek-2 compact system (bioMérieux). All isolates tested as not susceptible (i.e., intermediate or resistant) to meropenem (MEM) using Vitek-2 compact system confirmed by detecting the *bla*_KPC_ (GenBank accession numbers: MZ546615.1), *bla*_IMP_ (GenBank accession numbers: MZ673648.1), *bla*_VIM_ (GenBank accession numbers: NZ_CVVN01000111.1), *bla*_oprD2_ (GenBank accession numbers: NC_002516.2), *bla*_NDM_ (GenBank accession numbers: OL348380.1), *bla*_OXA-23_ (GenBank accession numbers: JN665073.1), *bla*_OXA-48_ (GenBank accession numbers: KX523902.1) and *bla*_OXA-58_ (GenBank accession numbers: KC004135.1) genes using individual laboratory-developed realtime PCR assays as previously described (Table S1). The PCR products were subjected to sequence analysis in both directions by Sangon Biotech Co., Ltd., Shanghai, China.

### Bacterial isolation using serum separator gel tubes.

The bacterium was separated from positive BCs as described previously (25) with little modification. Briefly, 2 mL of positive BC were transferred into a serum separator tube (SST) (BD, Franklin Lakes, NJ, USA) using a 2.5 mL syringe, and the bacterial cells were separated after centrifugation for 5 min at 3500 rpm (rpm) at room temperature. The supernatant was removed, and the bacterial cells were collected from the surface of the silicon layer of the SST and suspended in 1 mL sterile water. The bacterial suspension was transferred to a 1.5-mL microcentrifuge tube after mixing gently and centrifuged at 13,000 rpm for 1 min. The pellets were resuspended in 1 mL sterile water and centrifuged again at 13,000 rpm for 1 min. The supernatant was removed, and the pellets was retained for further analysis.

### DNA extraction.

The bacterial pellets were suspended in 300 μL sterile distilled water and heated to 100°C for 10 min. Subsequently, 450 μL of phenol-saturated water (pH 7.9; Sangon Biotech) was added to the lysate. The mixture was kept at room temperature for 2 min, followed by centrifugation (13000 rpm, room temperature, 2 min) in order to release DNA into the supernatant. Purification of bacteria genomic DNA of the supernatant was conducted with the Spin Column DNA Cleanup Minipreps Kit (Sangon Biotech) according to the manufacturer's instructions. The DNA quantity and quality were evaluated using the Nanodrop 2000 spectrophotometer. Extracted bacterial DNA was stored at −20°C until use.

### Microfluidic chip for parallel identification of multiple carbapenemase genes.

**FIG 2:**
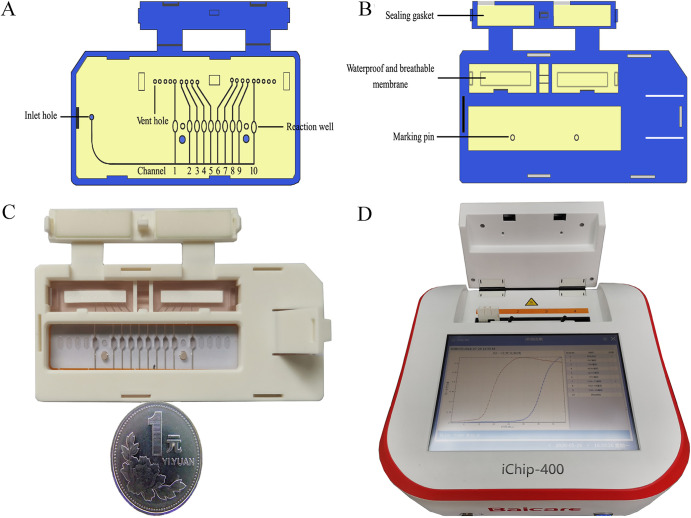
Microfluidic chip and setup for on-chip LAMP for the parallel detection of multiple carbapenemases genes. (A) Schematic illustration the basement of the microfluidic chip. Channel 1–10 represented: 1, positive control; 2, IMP; 3, VIM; 4, NDM; 5, oprD2; 6, KPC; 7, OXA-23; 8, OXA-48; 9, OXA-58; 10, negative control; (B) schematic illustration the cover of the microfluidic chip; (C) photograph of the microfluidic chip; (D) photograph of the setup for on-chip LAMP with a temperature-controlled system and a real-time fluorescent acquisition unit.

The microfluidic chip for parallel identification of multiple carbapenemase genes was developed via the following steps:

**(i) Chip fabrication.** The card-type microfluidic chip was consisted of an array of 10 reactions (positive control, negative control and 8 carbapenemase genes). The microfluidic chip was fabricated with polymethylmethacrylate (PMMA) materials and composed of 2 layers: the bottom layer consisted of reaction wells, a primary channel, an inlet hole and a vent hole, while the top layer consisted of waterproof and breathable membranes, a sealing gasket and a vent hole clip. Each reaction well was closely connected with the primary channel. Before chip integration, the primers targeting 8 carbapenemase genes (bla_KPC_, bla_IMP_, bla_VIM_, bla_oprD2_, bla_NDM_, bla_OXA-23_, bla_OXA-48_ and bla_OXA-58_) were designed and embedded into the reaction wells of the bottom layer. When the chip was heated, the reaction mixture was cut off by the waterproof and breathable membranes. In addition, the air pressure inside and outside of the chip was the same, thus isolating the reaction wells to reduce possible contamination among reaction wells (Fig. 2A to C). The primers for LAMP assays were listed in Table S2.

**FIG 3:**
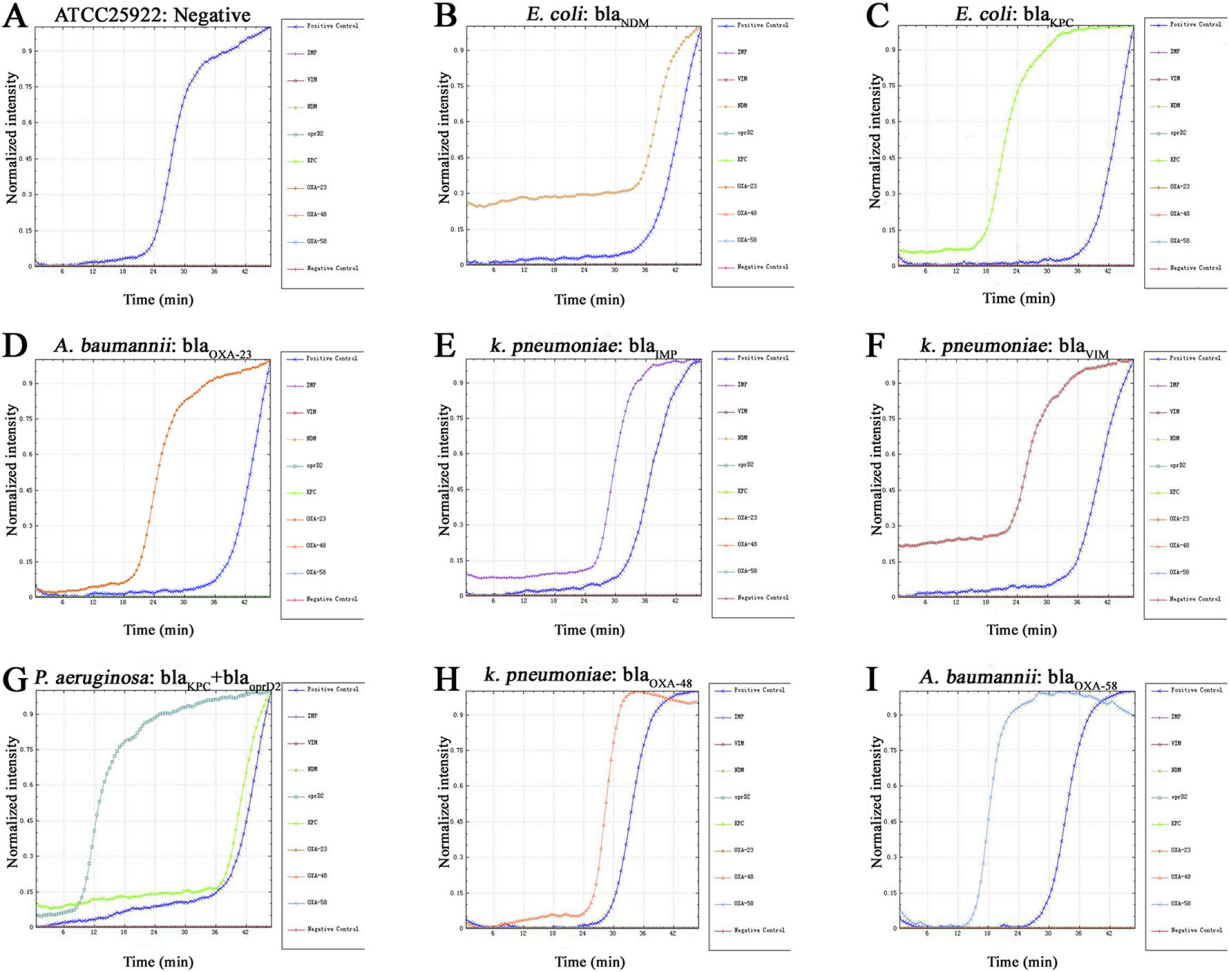
Amplification curves for simultaneous detection of multiple carbapenemase genes using on-chip LAMP. (A) ATCC25922, negative control for carbapenemase genes; (B) E. coli, bla_NDM_; (C) E. coli, bla_KPC_; (D) A. baumannii, bla_OXA-23_; (E) K. pneumoniae, bla_IMP_; (F) K. pneumoniae, bla_VIM_; (G) P. aeruginosa, bla_KPC_+ bla_oprD2_; (H) K. pneumoniae, bla_OXA-48_; (I) A. baumannii, bla_OXA-58_. The template used was gDNA extracted from clinical bacterial isolates with concentrations of 50 ng/μL.

**(ii) Real time on-chip fluorogenic LAMP Assay.** The isothermal amplification reaction solution was thawed at room temperature. Next, 58 μL of isothermal amplification reaction solution and 14 μL extracted nucleic acid specimen were added to the prepared 200 μL centrifuge tube and shaken gently to mix evenly. A micropipette was used to transfer 72 μL of the prepared mixture into the main channel of the chip through the inlet until the channel was completely full. Ultimately, the inlet and outlet holes were sealed completely with PMMA cover. The chip was placed into the nucleic acid analyzer (Fig. 2D), incubated at 37°C for 5 min, heated to 65°C, and then kept at a constant temperature of 65°C for 40 min before terminating the reaction. The nucleic acid analyzer can detect the increase in the fluorescence signal in real time, and exponentially increasing nucleic acid amplification curves are obtained. The specificity of our system was assessed using prepared gDNA samples with concentrations of 50 ng/μL, including the targeted 8 carbapenemase genes. The expected positive signals were recognized by typical sigmoidal amplification curves, and only the targeted carbapenemase genes showed positive results (Fig. 3).

### Statistical analyses.

Statistical analysis was performed using 95% confidence interval (95% CI) and calculated with VassarStats online software. The positive predictive value (PPV) and negative predictive value (NPV) of microfluidic chip-based LAMP amplification method of bacterial isolates and positive BCs were calculated using an aggregate gold standard as follows. For a positive result, the isolate must be meropenem intermediate or resistant and be confirmed by sequencing. For a negative result, the isolate must be meropenem sensitive or be confirmed by sequencing.
