# Supplementary material for: Development of Microfluidic Chip-Based Loop-Mediated Isothermal Amplification (LAMP) Method for Detection of Carbapenemase Producing Bacteria
Source: Microbiol Spectr. 2022 Aug 18;10(5):e00322-22. doi: 10.1128/spectrum.00322-22 (PMC9603548; doi:10.1128/spectrum.00322-22)
Supplement: Supplemental file 1 — Tables S1-S6. Download spectrum.00322-22-s0001.pdf, PDF file, 0.2 MB [file spectrum.00322-22-s0001.pdf]

Supplemental Tables

Table S1. Primer sequences used for quantitative PCR assays.

Genes	Primers sequence (5′ → 3′)		Reference
KPC	Forward primer	TCGCTAAACTCGAACAGG	(1)
	Reverse primer	TTACTGCCCGTTGACGCCCAATCC	
VIM	Forward primer	GTTTGGTCGCATATCGCAAC	(1)
	Reverse primer	AATGCGCAGCACCAGGATAG	
IMP	Forward primer	GGAATAGAGTGGCTTAAYTCTC	(2)
	Reverse primer	CCAAACYACTASGTTATCT	
NDM	Forward primer	GGTTTGGCGATCTGGTTTTC	(2)
	Reverse primer	CGGAATGGCTCATCACGATC	
OXA-48	Forward primer	GCGTGGTTAAGGATGAACAC	(2)
	Reverse primer	CATCAAGTTCAACCCAACCG	
oprD2	Forward primer	ATGAAAGTGATGAAGTGGAGCG	(3)
	Reverse primer	TTACAGGATCGACAGCGGATAG	
OXA-23	Forward primer	CCTCAGGTGTGCTGGTTATT	(4)
	Reverse primer	ATGTAGAGGCTGGCACATATTC	
OXA-58	Forward primer	ATACTCTCACTGAGGCAGGTTGG	(5)
	Reverse primer	CTGTCCCAATGATCACTTGCAA	

Table S2. Primer sequences used for LAMP used in this study.

Genes	Primers sequence (5′ → 3′)	
IMP	IMP-F3	GCAGAGTCTTTGCCAGCG
	IMP-B3	GTCGCTCGGAAACGGAGAGG
	IMP-FIP	CCGGTTTAGGAACAACGCCCCTGAAAAGCTTGCGGAAGGC
	IMP-BIP	TGACACTCCCGTTACGGCTAAAGTTCGAGCCACGCTCCACA
	IMP-LB	ACCCGTTAACTTCTTCAAACGAAG
VIM	VIM-F3	GTGCTTTGACAACGTTCGC
	VIM-B3	CGACGTCCCGTCAGCGAA
	VIM-FIP	AAGCAGAGGTCGTCCGTCCCGTGTGTGCTGGAGCAAGTCT
	VIM-BIP	GCCCGTCAGCCAGCGCGGCCGGGTTGTGCCGTTCCGGAGTT
	VIM-LF	TTCCCCGCAGACGTGCTTG
NDM	NDM-F3	TTGCACTGGCGGTTTCC
	NDM-B3	GCCGGCGTCCGAGGTGGTG
	NDM-FIP	CGGAACCCCTTCGCTTCGGCCAGGTAGCACTCAGTTCGC
	NDM-BIP	CCTGCTGCTCCGCAACTACTCGAGGAAGCCTTGGGTCCA
	NDM-LF	TCAACCGTGACGGCAAGA
oprD	oprD-F3	TTGCACTGGCGTATTCC
	oprD-B3	GCCGGATTCATAGGTAGTG
	oprD-FIP	ATGAACCCCTTCGCTTGGGCCAGGTAGCACTCAGTTCGC
	oprD-BIP	CCTGCTGCTCCGCAACTACTATAGGAAGCCTTGGGTCCA
	oprD-LB	TCAACCGTTACGGCAAGA
	oprD-LF	TGATCGCTGACGAATGCGT
KPC	KPC-F3	GGCAGCAGTTTGTTGCGTGG
	KPC-B3	CGCTGTGCTTGTCCGCCT
	KPC-FIP	GGTTTTGTCTCCGACTGCCCACTAAAGGGAAACACGACCGG
	KPC-BIP	TGGCACGGCAACGGACTCGGCCGACGGCCAACACACGAGGTG
OXA-23	OXA23-F3	CAGACGCGGTGCCAGCCTCT
	OXA23-B3	CGCGACGTCGCGCAAGTT
	OXA23-FIP	TGACCTTTTCTCGCCCTTCCCGGTTGACGGCCCTGCGCGGA

	OXA23-BIP	CCGCTTGGGAAAAAGACCGGACACCCTGCGAGACTGGGACTGC
	OXA23-LB	AGGAGAAGCCCGGAAGCTTTC
OXA-48	OXA48-F3	GCGTCGCGGACGGCCTGCG
	OXA48-B3	TGTTCCGCCTTAACCACGC
	OXA48-FIP	GCACAACTACGCCCTGTGCGTTCGGTAGCAAAGGACGGGCAAGA
	OXA48-BIP	TAAACGGGCGAACCAAGCCGGGCGCGCAAGCTCGTGGGA
	OXA48-LF	GTTCAGTAAAGTGAGCCGTCCAAC
OXA-58	OXA58-BIP	AGAGCGCAGAGGGGAGAATC-CACTTGCGGGTCTACAGC
	OXA58-LF	GAAAGGGCCTTTGACAATTACACC
	OXA58-LB	ATGCTAAAAGTGGCTGGGGAAT
	OXA58-BIP	AGAGCGCAGAGGGGAGAATC-CACTTGCGGGTCTACAGC
Positive Control	PC-F3	ATCTCACACTCATGGCAG
	PC-B3	CCAAAACTGCAGCTTGG
	PC-FIP	AAGCTCAAGAACAACCCGTCCCCCCTTCCACATTCCACTT
	PC-BIP	GTGCTAGGAAGCCGTTTTTTTATGCCCCCTGTAGAGAACATTCTT
	PC-LF	GTAATAATGACTTAATGGAA
	PC-LB	TGGCATCCTTTCATATGTTC

Table S3. Carbapenemase misclassified strains by the microfluidic chip-based LAMP amplification method in retrospective study.

Organism (n)	Reference method (culture plus sequencing)	Microfluidic chips
K.pneumoniae (1)	NA	KPC
K.pneumoniae (1)	NA	oprD2
K.pneumoniae (1)	KPC	Negative
K.pneumoniae (1)	KPC	KPC+OXA-23
K.pneumoniae (2)	KPC	NDM
K.pneumoniae (1)	IMP+KPC	NDM+KPC
K.pneumoniae (1)	IMP	KPC
E. coli (2)	KPC	KPC+NDM
E. coli (1)	NDM	KPC+NDM
E. coli (2)	NDM	KPC
E. coli (1)	NDM	IMP
K.pneumoniae (1)	NDM	oprD2+NDM
K.pneumoniae (2)	NDM	KPC+NDM
A. baumannii (1)	OXA-23	OXA-23+NDM
A. baumannii (1)	OXA-23	Negative
A. baumannii (2)	OXA-23+KPC	OXA-23+KPC+NDM
A. baumannii (2)	OXA-23+NDM	OXA-23+KPC+NDM
A. baumannii (1)	OXA-23+KPC+OXA-48	OXA-23+KPC+OXA-48+OXA-58

Table S4. Summary of the results of the microfluidic chip-based LAMP amplification method compared to the truth consisting of conventional molecular (Sequencing) carbapenemase detection tests in retrospective study.

	Organism	TP (%)	TN (%)	FP (%)	FN (%)	Total
Enterobacteriaceae	*K.pneumoniae*	37 (55.2)	19 (28.4)	10 (14.9)	1 (1.5)	67
	E. coli	16 (32.0)	28 (56)	6 (12.0)	0 (0.0)	50
	E. cloacae	2 (100.0)	0 (0.0)	0 (0.0)	0 (0.0)	2
	C. Koseri	2 (33.3)	4 (66.7)	0 (0.0)	0 (0.0)	6
**Non-fermentative**	*A. baumannii*	29 (43.3)	31 (46.3)	6 (8.9)	1 (1.5)	67
	Total	86 (44.8)	82 (42.7)	22 (11.5)	2 (1.0)	192

TP: True positive; FP: False positive; TN: True negative; FN: False negative

Table S5. Detection performance of microfluidic chip-based LAMP amplification method for single carbapenemase, multiples carbapenemase and carbapenemase negative bacteria.

Bacteria	Total	Accuracy [95% CI]
Single carbapenemase	79	80.0 [69.5-87.2]
Enterobacteriaceae	58	
Non-fermentative	21	
Multiples carbapenemase	29	79.3 [61.3-90.5]
Enterobacteriaceae	14	
Non-fermentative	15	
Carbapenemase negative	84	98.8 [92.8-100.0]
Enterobacteriaceae	53	
Non-fermentative	31	

Table S6. Results from the microfluidic chip-based LAMP amplification method (directly isolate from BCs) and the reference method (culture plus sequencing) [a].

Organism (n)	Microfluidic chips	Reference method (culture plus sequencing)	Outcome
K.pneumoniae (1)	KPC	NA	FP
E. coli (1)	KPC	NA	FP
E. coli (1)	oprD2	NA	FP
A. baumannii (1)	OXA-23	NA	FP
E. coli (5)	KPC	KPC	TP
E. coli (1)	KPC+NDM	KPC+NDM	TP
E. coli (1)	KPC+oprD2	KPC	FP
K.pneumoniae (5)	KPC	KPC	TP
K.pneumoniae (1)	KPC+NDM	KPC	FP
P. mirabilis (1)	KPC	KPC	TP
A. baumannii (1)	OXA-23+OXA-48+KPC	OXA-23+KPC	FP
A. baumannii (2)	OXA-23+NDM	OXA-23+NDM	TP
A. baumannii (1)	OXA-23+OXA-48+NDM	OXA-23+NDM	FP

[a] True-positive and false-positive results are shown

TP: True positive; FP: False positive; NA, Not applicable

Supplemental References

1. Monteiro J, Widen RH, Pignatari AC, Kubasek C, Silbert S. 2012. Rapid detection of carbapenemase genes by multiplex real-time PCR. J Antimicrob Chemother 67: 906-909. https://doi.org/10.1093/jac/dkr563.
2. Liu Q, Liu L, Li Y, Chen X, Yan Q, Liu WE. 2019. Fecal Carriage and Epidemiology of Carbapenem-Resistant Enterobacteriaceae Among Hospitalized Patients in a University Hospital. Infect Drug Resist 12:3935-3942. https://doi.org/10.2147/IDR.S233795.
3. Shen J, Pan Y, Fang Y. 2015. Role of the Outer Membrane Protein OprD2 in Carbapenem-Resistance Mechanisms of Pseudomonas aeruginosa. PLoS One 10:e0139995. https://doi.org/10.1371/journal.pone.0139995.
4. Teo JW, La MV, Lin RT. 2016. Development and evaluation of a multiplex real-time PCR for the detection of IMP, VIM, and OXA-23 carbapenemase gene families on the BD MAX open system. Diagn Microbiol Infect Dis 86:358-361. https://doi.org/10.1016/j.diagmicrobio.2016.08.019.
5. Girlich D, Bonnin RA, Bogaerts P, De Laveleye M, Huang DT, Dortet L, Glaser P, Glupczynski Y, Naas T. 2017. Chromosomal Amplification of the blaOXA-58 Carbapenemase Gene in a Proteus mirabilis Clinical Isolate. Antimicrob Agents Chemother 61:e01697-16. https://doi.org/10.1128/AAC.01697-16.
